# Artificial Intelligence and Colposcopy: Automatic Identification of Vaginal Squamous Cell Carcinoma Precursors

**DOI:** 10.3390/cancers16203540

**Published:** 2024-10-20

**Authors:** Miguel Mascarenhas, Inês Alencoão, Maria João Carinhas, Miguel Martins, Tiago Ribeiro, Francisco Mendes, Pedro Cardoso, Maria João Almeida, Joana Mota, Joana Fernandes, João Ferreira, Guilherme Macedo, Teresa Mascarenhas, Rosa Zulmira

**Affiliations:** 1 Department of Gastroenterology, São João University Hospital, 4200-319 Porto, Portugal; 2 WGO Gastroenterology and Hepatology Training Center, 4200-427 Porto, Portugal; 3 Faculty of Medicine, University of Porto, 4150-180 Porto, Portugal; 4 Department of Gynecology, Centro Materno-Infantil do Norte Dr. Albino Aroso (CMIN), Santo António University Hospital, 4099-001 Porto, Portugal; inesalencoao@gmail.com (I.A.);; 5 Department of Mechanical Engineering, Faculty of Engineering, University of Porto, 4150-180 Porto, Portugal; 6 Department of Gynecology, São João University Hospital, 4200-319 Porto, Portugal

**Keywords:** vaginal neoplasms, HSIL, LSIL, colposcopy, artificial intelligence

## Abstract

**Background/Objectives**: While human papillomavirus (HPV) is well known for its role in cervical cancer, it also affects vaginal cancers. Although colposcopy offers a comprehensive examination of the female genital tract, its diagnostic accuracy remains suboptimal. Integrating artificial intelligence (AI) could enhance the cost-effectiveness of colposcopy, but no AI models specifically differentiate low-grade (LSILs) and high-grade (HSILs) squamous intraepithelial lesions in the vagina. This study aims to develop and validate an AI model for the differentiation of HPV-associated dysplastic lesions in this region. **Methods**: A convolutional neural network (CNN) model was developed to differentiate HSILs from LSILs in vaginoscopy (during colposcopy) still images. The AI model was developed on a dataset of 57,250 frames (90% training/validation [including a 5-fold cross-validation] and 10% testing) obtained from 71 procedures. The model was evaluated based on its sensitivity, specificity, accuracy and area under the receiver operating curve (AUROC). **Results**: For HSIL/LSIL differentiation in the vagina, during the training/validation phase, the CNN demonstrated a mean sensitivity, specificity and accuracy of 98.7% (IC95% 96.7–100.0%), 99.1% (IC95% 98.1–100.0%), and 98.9% (IC95% 97.9–99.8%), respectively. The mean AUROC was 0.990 ± 0.004. During testing phase, the sensitivity was 99.6% and 99.7% for both specificity and accuracy. **Conclusions**: This is the first globally developed AI model capable of HSIL/LSIL differentiation in the vaginal region, demonstrating high and robust performance metrics. Its effective application paves the way for AI-powered colposcopic assessment across the entire female genital tract, offering a significant advancement in women’s healthcare worldwide.

## Simple Summary

A colposcopy provides a comprehensive examination of the female genital tract, including the cervix, vagina, and vulva. However, its diagnostic accuracy, especially for vaginal lesions, remains suboptimal due to the challenge in detecting subtle alterations. Integrating artificial intelligence (AI) into colposcopy holds potential to enhance the detection rates of clinically important lesions. Our study pioneered the development of an AI algorithm capable of differentiating low-grade (LSILs) and high-grade (HSILs) squamous intraepithelial lesions in the vagina. The promising results we achieve in differentiating HPV-associated dysplastic lesions demonstrate that AI can significantly address the current challenges in medical practice. There are already promising results when using AI to detect HPV lesions in the cervix and anus, suggesting that in the future, this ubiquitous tool for lesion detection across different anatomical regions could be a reality. The future integration of these technologies into the colposcopy process could revolutionize healthcare, making early detection a reality for women worldwide.

## 1. Introduction

Infection by the human papillomavirus (HPV) constitutes the most frequent sexually transmitted infection. Persistent infection by high-risk HPV types (most frequently HPV 16) is linked to the occurrence of anogenital neoplasia, most notably cervical and anal cancer [1]. The presence of dysplastic lesions of the vagina, despite being less frequent than their counterparts, shares the same carcinogenesis process, and an increase in the detection of precursor lesions has been registered in recent years [2].

Diagnosing these lesions poses a complex challenge due to their tendency to be multifocal [3]. Additionally, compared to the cervix, there is a weaker correlation between vaginoscopy patterns and histopathological classification. Moreover, concerns have been raised regarding the suboptimal accuracy and high intra/interobserver variability of vaginoscopy [4]. The lower prevalence of vaginal cancers and precursor lesions combined with the inexistence of standardized screening approaches constitutes a hurdle for early diagnosis [5].

Differentiating between vaginal low-grade and high-grade squamous intraepithelial lesions (LSILs and HSILs, respectively) is pivotal to select the most appropriate management and prevent unnecessary treatments [6]. Indeed, approximately 50% of LSILs have the potential to regress. In contrast, HSILs are true precursor lesions and may progress to invasive squamous carcinoma, which occurs in about 12% of patients in whom an HSIL is identified. Therefore, the suboptimal diagnostic yield of the currently existing diagnostic techniques has prompted research for potential technological solutions [4].

Recently, there has been significant attention on artificial intelligence (AI). Advances in computer performance have facilitated the successful development of deep learning models, particularly applied in medical fields involving image and video analysis. Convolutional neural networks (CNNs) are a deep learning approach specifically employed to recognize and classify image patterns. Inspired by the neurobiological synapse process, these algorithms replicate it by capturing spatial hierarchies and patterns in an efficient manner [7].

In the perineal region, most primary AI research has primarily focused on accurately detecting and differentiating HSILs from LSILs in the cervix and anus during colposcopy and anoscopy procedures, respectively [8,9,10,11,12,13,14,15,16]. These advancements have the potential to improve diagnostic accuracy, standardize evaluations, and reduce interobserver variability. However, it is important to note that developing a CNN specifically trained for the cervix or anus does not ensure comparable accuracy when applied to the vagina. Due to the multifocal nature of HPV infection, lesions can also appear, and as of our knowledge, there are no published works addressing the development of this type of AI algorithm in this area of the body.

The aim of this study is to develop and validate a CNN to automatically differentiate vaginal HSILs from LSILs using images retrieved from vaginoscopy exams.

## 2. Materials and Methods

A retrospective study with a non-interventional design was performed using colposcopies performed at a single tertiary center (Centro Materno Infantil do Norte) in Porto, Portugal, between December 2022 and December 2023 (one year). A total of 57,250 frames of vaginal walls were collected and included in the dataset to develop, train, and validate the CNN-based model. This study was approved by the ethical committee (IRB 2023.157 (131-DEFI/123-CE)), in accordance with the principles indicated in the Declaration of Helsinki.

All colposcopies were performed by a single expert according to the current standard of care and using the same Zeiss colposcope FC 150 (Oberkochen, Germany). Each procedure was separated into four stages, including initial non-stained observation, followed by assessment with 3% acetic acid, and then with lugol. The fourth stage consists of the fact that the procedure may involve the performance of therapeutic manipulation (e.g., laser ablation). Frames from these four stages were included in the dataset. Note that each procedure may comprise any combination of these four stages. Biopsies of suspected lesions were performed, and the collected tissue was preserved. The video segments of the observation of vaginal walls were retrieved, and, using VLC Media Player (VideoLan, Paris, France), the collected videos were segmented in still frames.

**Figure 1:**
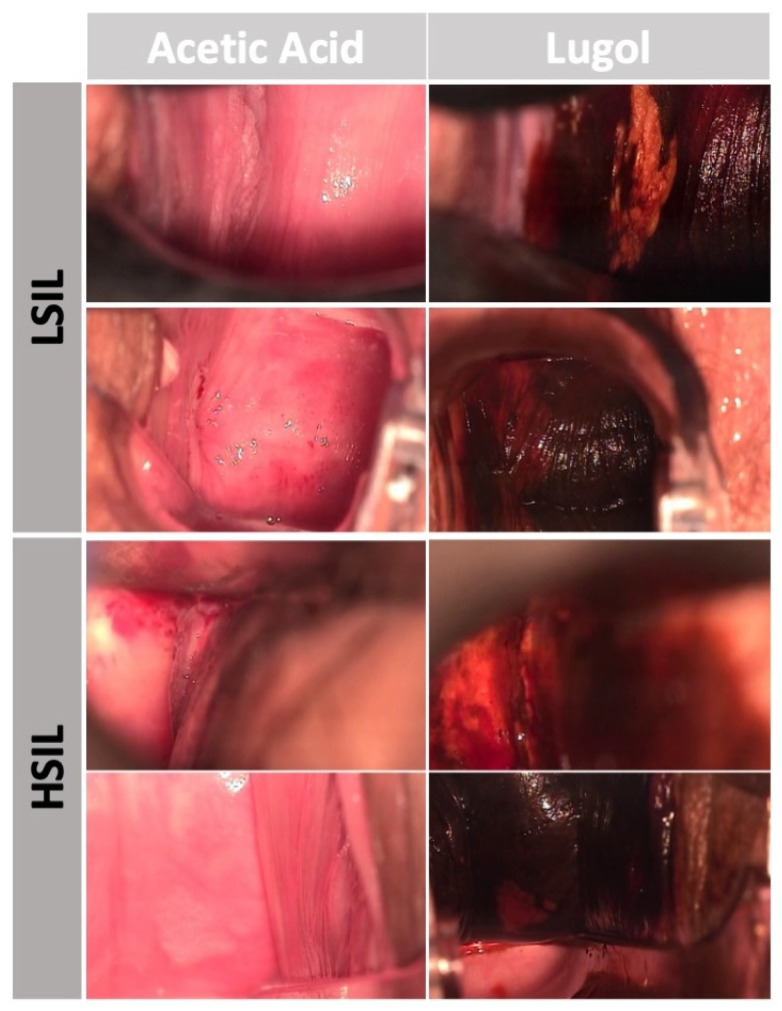
Examples of LSIL and HSIL frames (with acetic acid or lugol staining) during assessment of the vagina during colposcopy.

A total of 71 vaginoscopy procedures were ultimately included and a dataset of 57,250 frames was assessed. From the collected frames, 25,455 were classified as HSILs, and the remaining 31,795 frames were categorized as LSILs. The biopsy histopathology report from the vaginoscopy evaluation of the observed lesion was consistently referenced to classify each corresponding frame as either an HSIL or LSIL (Figure 1).

**Figure 2:**
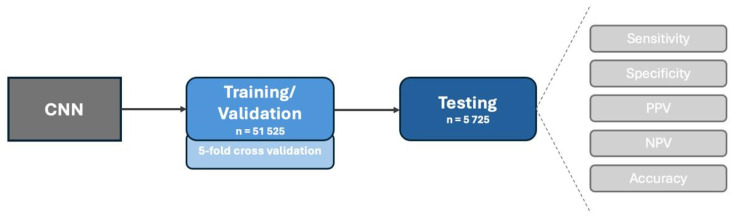
Design of the study. The data were divided into a training/validation set (90% of total dataset) and a testing set (the remaining 10%). During training/validation, a 5-fold cross-validation was performed to assess the robustness of the model. The primary outcomes of this study were sensitivity, specificity, positive and negative predictive value (PPV and NPV, respectively) and accuracy.

The dataset was split into training/validation (90%) and testing (10%) datasets, corresponding to 51,525 frames and 5725 frames, respectively. The testing set was used to independently validate the performance of the CNN. No patient split was performed to maximize data use. The flowchart in Figure 2 describes the study’s methodology.

The CNN was created based on a ResNet10 model pre-trained on ImageNet, a vast image dataset for object recognition [17]. ResNet10 is designed to extract features from input images efficiently, consisting of multiple convolutional layers, batch normalization layers, and residual connections. The model was custom-tailored for our classification task (HSIL vs. LSIL), replacing the original classification layer with a custom classification head containing a fully connected layer followed by an activation function. This new layer mapped the extracted features to the number of classes and converted the scores into log probabilities, assuring numerical stability during training with the negative log-likelihood loss. Hyperparameters including learning rate (0.0001), batch size (128), and epoch number (10), were fine-tuned through trial and error. Data preparation utilized libraries including FFMPEG, Pandas, and Pillow, while the model was implemented in PyTorch. Also, no data augmentation techniques were used. The computational system included dual NVIDIA Quadro RTXTM 80000 GPUs (NVIDIA Corp, Santa Clara, CA, USA) and an Intel 2.1 GHz Xeon Gold 6130 processor (Intel, Santa Clara, CA, USA).

**Figure 3:**
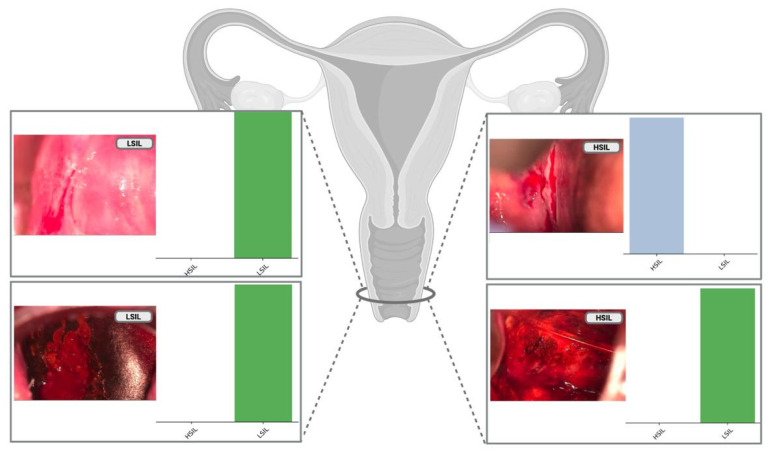
Examples of the algorithm’s prediction for whether a lesion was an HSIL (high-grade intraepithelial squamous lesion) or an LSIL (low-grade intraepithelial squamous lesion). Every frame was assigned to the category with the highest calculated probability. These classifications, provided by the convolutional neural network (CNN), were compared to the histopathological classification (shown in the upper left corner), considered the gold standard.

The model assigned a probability to each frame to be classified as either an HSIL or LSIL. Each frame’s final classification was based on the category with the higher probability. The model’s classifications were then compared to the gold standard, the corresponding histopathological diagnosis (Figure 3).

We performed a 5-fold cross-validation to assess the robustness of the CNN in the training/validation stage. The training/validation dataset, comprising 90% of the total data, was split into five equal-sized folds, using a class-stratified division. We carried out five different iterations in total. In each iteration, four folds were used to train the model, while the fifth fold was used for validation. The particular folds employed for training and validation in the CNN varied throughout each iteration. After each iteration, sensitivity, specificity, accuracy, positive predictive value (PPV), and negative predictive value (NPV) we determined. Additionally, the area under the conventional receiver operating characteristic curve (AUC-ROC) for each iteration was also calculated. The inputs from each iteration of the training/validation phase allowed for fine-tuning of specific hyperparameters, which were subsequently evaluated using the test dataset. During this stage, the remaining 10% of the data were used to independently assess the CNN’s performance. The statistical analysis was performed using Sci-Kit Learn v0.22.2 [18].

## 3. Results

The AI model was trained and tested using a dataset of 57,250 frames (25,455 HSILs and 31,795 LSILs, all confirmed through histopathological analysis). Of this dataset, 90% (57,250 frames) was utilized during the training/validation phase, while the remaining was used during testing phase. The number of frames, patients and lesions (HSILs and LSILs) include in each iteration of the five-fold cross-validation, during the training and testing phase, are detailed in Table 1.

**Figure 4:**
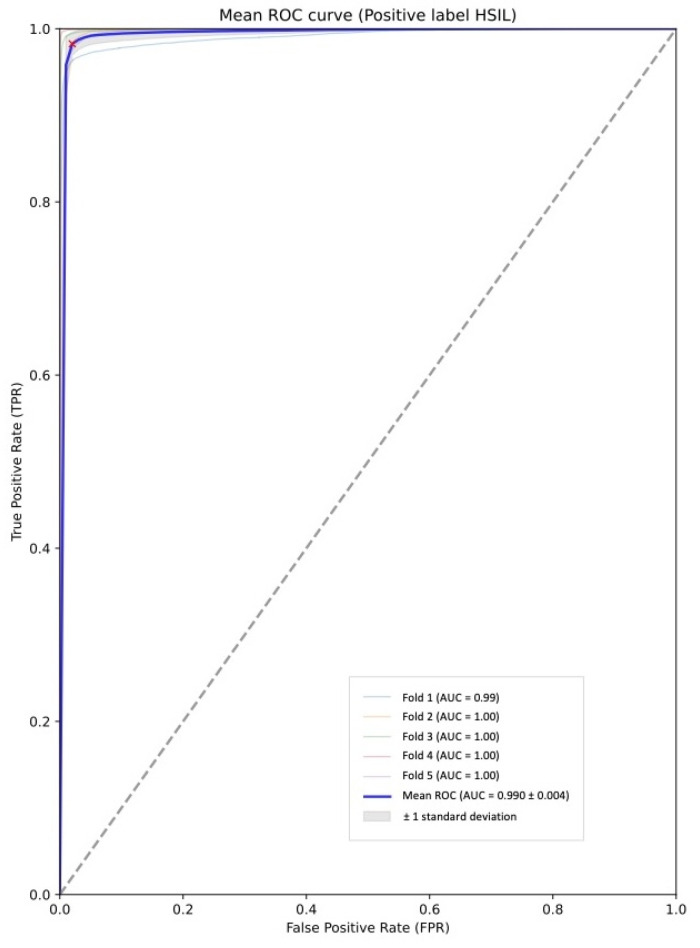
Area under the conventional receiver operating characteristic curve (AUC-ROC) for each iteration during 5-fold cross-validation.

During the training/validation phase, the analysis of five-fold cross-validation revealed a median sensitivity and specificity of 98.7% (IC95% 96.7–100.0%) and 99.1% (IC95% 98.1–100.0%), respectively. The median PPV was 98.9% (IC95% 97.6–100.0%) and NPV was 98.9% (IC95% 97.4–100.0%). The overall median accuracy was calculated at 98.9% (IC95% 97.9–99.8%). The mean AUROC was 0.990 ± 0.004 (Figure 4). Table 2 displays the diagnostic performance metrics of each iteration.

During the test phase, the CNN demonstrated a sensitivity of 99.6% and specificity of 99.7%, achieving an overall accuracy of 99.7%. The PPV and NPV were 99.6% and 99.7%, respectively. The F1 score was 99.6%.

## 4. Discussion

To our knowledge, this is first proof-of-concept CNN developed for HPV-dysplastic lesion detection and differentiation in the vaginal region. The AI model effectively discriminated HSILs and LSILs within vaginal frames, demonstrating exceptional sensitivity (99.6%) and specificity (99.7%). Such an advancement could contribute to improved colposcopic assessment of female genital tract, increasing the detection rates of clinically important lesions and enhancing the overall cost-effectiveness of the procedure.

High-resolution colposcopic evaluation of the genital tract is a procedure that requires significant expertise. On the one hand, it is important to detect any deviations from the normal mucosal pattern. On the other, it is necessary to distinguish lesions that require treatment from those that can be monitored. This complexity can result in some lesions being overlooked and others being overtreated. Using a colposcope (with high resolution and magnification) facilitates easier and more accurate visualization of the cervical region and also allows for examination of the vaginal and vulvar areas. This comprehensive evaluation of the genital tract is the primary advantage of this device, considering that the effects of HPV can be multifocal, although mastering its use requires significant time and training [19].

The development and implementation of deep learning models in imaged-based specialties has rapidly accelerated in recent times, and these models may be become increasingly indispensable tools for diagnostic and therapeutic procedures. Current AI models for gynecological assessment using high-resolution colposcopy are still in their early phase and primarily limited to the cervix [20]. However, considering the complexity of the female genital tract and fundamental data science principles, it is improbable that cervical trained models can accurately detect and differentiate lesions in vaginal walls. While intuitively appealing due to the comprehensive nature of colposcopy, this approach would not be practical.

Therefore, this AI model marks an important milestone, being the first worldwide specific AI model developed for the vaginal area. The rigorous inclusion of only biopsied lesions for training the model brings it closer to the ground truth, mitigating the risk of suboptimal training of the CNN. In addition, the model demonstrated high performance metrics not only for the test set, but also during 5-fold cross-validation of the training set. This is a particularity worth mentioning, as it highlights the model’s consistent efficiency in distinguishing HSIL and LSIL frames, regardless of how frames are distributed. This work is significant not only due to its pioneering nature but also because it highlights the development of a robust AI model that proficiently differentiates HSILs from LSILs, regardless of frame distribution. Compared to the literature that reports a 60% overall accuracy of colposcopy in detecting VAIN lesions, it can be inferred that the development and integration of AI-enhanced colposcopy/vaginoscopy has the potential to substantially improve diagnostic accuracy and positively impact women’s health [21,22].

Additional strengths of this study include the incorporation of still frames of the entire procedure, encompassing non-stained, stained and subsequent manipulated frames via biopsy or laser treatment. This comprehensive dataset exposes the model to a diverse range of clinically relevant visual information, including tissue alterations and blood presence. Moreover, the model was also trained on HPV-related lesions situated in different locations on vaginal walls with different angles of visualization. This expanded exposure also enhances the model’s clinical utility by rendering it more adaptable to real-world clinical practice.

It is important to acknowledge some limitations of this methodology. First, we used still frames from procedures performed with colposcopes in the vaginal region, employing a non-annotation labelling methodology. This required the exclusion of complex images containing more than one lesion (e.g., different histologies within the same frame) to prevent model mis-training. Additionally, when evaluating certain vaginal areas, it may be necessary to isolate specific regions using tools (e.g., Kelly forceps) for accurate assessment. As a result, frames containing both the lesion and tool simultaneously had to be excluded, reducing the number of usable frames. This reduction in data could increase the risk of model overfitting, potentially making the model less effective in real clinical settings. Moreover, the absence of patient split between the training and testing sets poses a risk of data leakage, as similar frames may have appeared in both groups, potentially inflating the model’s performance metrics. Despite these challenges, we took steps to mitigate these risks by closely monitoring the model’s performance on the validation and test sets to ensure an unbiased evaluation.

This study is retrospective and was conducted at a single center, with a relatively small amount of data. Consequently, the possibility of demographic bias (selection bias) cannot be excluded, which may further impact the external validity of the findings and suggest that performance accuracy may differ in real-word clinical settings with diverse populations. Moreover, since this model is based on data capture from only one type of device, we cannot guarantee its interoperability, which is currently an essential characteristic for models to be considered true AI. Despite these limitations, technological advancements in healthcare often rely on incremental progress, which should be shared and acknowledged. This study serves as a proof of concept (technological readiness level 3, one step behind the current level of corresponding cervical AI models), demonstrating that AI models for these vaginal regions can indeed be developed with adequate data and rigorous methodological techniques, although prospective and multicentric studies are still needed to validate this.

## 5. Conclusions

To conclude, this AI algorithm demonstrated great promise in detecting and differentiating low-grade and high-grade squamous intraepithelial lesions (LSILs and HSILs, respectively) in the vagina. This is the first AI model specifically developed and trained for this area. However, further investigation is needed to evaluate broader applicability. While this study is preliminary, retrospective, and conducted at a single center, utilizing still frames, which may affect the generalizability of the results, the model’s performance is a positive first step. Challenges such as potential selection bias and overfitting must be addressed in future investigations to fully assess its generalizability and clinical utility. This model represents the initial step towards a comprehensive, AI-powered colposcopic assessment encompassing the entire female genital tract.

## Figures and Tables

**Table 1:** Distribution of frames, patients and lesion types across groups. The data were divided into a training/validation set (90% of total dataset) and a testing set (the remaining 10%).

		HSILs		LSILs	
		Frames	Patients	Frames	Patients
Training/Validation phase	Fold 1	4582	18	5723	18
	Fold 2	4582	17	5723	18
	Fold 3	4582	17	5723	18
	Fold 4	4582	18	5723	18
	Fold 5	4582	17	5723	18
Testing phase	Test set	2545	17	3180	18

**Table 2:** The dataset was divided into 90% for training and 10% for testing. We applied a stratified 5-fold cross-validation technique on the training set, randomly selecting 20% for validation, and maintained the class distribution. Each iteration consisted of training the model with 80% of the training set and evaluating the performance with the validation set. The procedure was repeated five times with different combinations. Performance metrics were evaluated on the validation fold during every iteration. The final result was averaged, providing a more robust evaluation of the model performance and reducing the risk of overfitting.

	Sensitivity (%)	Specificity (%)	PPV (%)	PPN (%)	Accuracy (%)	F1 Score (%)
Fold 1	100.0	97.9	97.5	100.0	98.8	98.7
Fold 2	99.6	99.7	99.6	99.7	99.6	99.6
Fold 3	97.2	99.6	99.8	97.8	98.6	98.5
Fold 4	99.8	99.4	99.2	99.8	99.6	99.4
Fold 5	96.8	98.5	98.1	97.5	97.7	97.3
Mean	98.7	99.1	98.8	98.9	98.9	98.7
IC 95%	96.7–100.0	98.1–100.0	97.6–100.0	97.3–100.0	97.9–99.8	97.6–99.8

## Data Availability

Non-identifiable data will be made available upon reasonable request.
